# Effects of Driving Task Demands and Information Load on AR-HUD Cognitive Efficiency: The Moderating Role of Working Memory Capacity in a VR-Based Simulated Driving Environment

**DOI:** 10.3390/jemr19030048

**Published:** 2026-05-03

**Authors:** Jing Li, Min Lin, Xinyu Feng, Hua Zhang, Chuchu Wang, Yulian Ma

**Affiliations:** 1 College of Furnishings and Industrial Design, Nanjing Forestry University, Nanjing 210037, China; linmin@njfu.edu.cn (M.L.); fengxinyunjfu@njfu.edu.cn (X.F.); 2784534015@njfu.edu.cn (H.Z.); wcc15225096621@163.com (C.W.); mayulian@njfu.edu.cn (Y.M.); 2 Jiangsu Co-Innovation Center of Efficient Processing and Utilization of Forest Resources, Nanjing Forestry University, Nanjing 210037, China

**Keywords:** augmented reality head-up display, working memory capacity, driving scenario, information load, cognitive efficiency, eye-tracking technology, VR-based simulation

## Abstract

The driving scenario and information load jointly influence the cognitive efficiency of augmented reality head-up display (AR-HUD) interfaces. However, the moderating role of drivers’ working memory capacity (WMC) remains unclear. To investigate this mechanism, a simulated driving experiment with a mixed design was conducted in a low-immersivity desktop virtual reality (VR) environment. First, 40 volunteers were screened using an automated operation span task, yielding 16 high- and low-WMC participants. They then drove under three scenarios (urban intersection, expressway, construction zone) and six levels of AR-HUD visual information load. Generalized linear models were applied to the reaction time, fixation duration, and pupil diameter. The results revealed a significant three-way interaction among WMC, scenario, and information load. High-WMC drivers maintained faster responses and lower subjective loads up to Levels 4–6, adopting a deep processing strategy; low-WMC drivers already showed cognitive overload at Level 4 and above, requiring an optimal load range of Level 2–3. The construction zone induced the steepest increase in cognitive load, whereas the expressway markedly reduced sensitivity to additional visual information. Therefore, the optimal AR-HUD information load must be adapted to drivers’ WMC: high-WMC drivers can safely handle Levels 4–6 in low- or medium-demand scenarios, whereas low-WMC drivers require a minimalist presentation of Levels 2–3 in high-demand situations. This study provides quantitative, empirically grounded guidelines for designing cognitively adaptive AR-HUD interfaces.

## 1. Introduction

An augmented reality head-up display (AR-HUD) directly superimposes virtual elements such as navigation, speed, and collision warnings onto the real road, allowing drivers to obtain information without shifting their gaze away from the forward view, thereby reducing the gaze deviation time and enhancing driving safety [1,2]. However, this “visual advantage” also introduces new cognitive risks: the superimposed information itself constitutes additional visual input, and, when the road scene is complex or the HUD content is overloaded, the total visual information may exceed the driver’s instantaneous processing capacity, leading to a significant increase in cognitive load and a decline in driving performance [3,4].

The amount of visual information results from two concurrent sources—the inherent visual–cognitive complexity of the driving scenario (e.g., highway vs. urban intersection) [5] and the information load of the AR-HUD interface (number of elements and update frequency). Their combination causes the same road segment to vary across low, medium, and high information levels, which directly governs AR-HUD cognitive performance. The individual working memory capacity (WMC) imposes a limit regarding how much information can be actively maintained and processed: high-WMC drivers retain low subjective loads and respond quickly even when information is abundant, whereas low-WMC drivers show earlier gaze fragmentation, pupil dilation, and delayed reactions [6]. Thus, the driver WMC, driving scenario, and AR-HUD information load jointly determine the cognitive efficiency of AR-HUD-assisted driving.

Therefore, this study employs eye-tracking technology to examine the interactive effects of the WMC, driving scenario, and information load on driving behavior and cognitive performance. It aims to inform the design of adaptive AR-HUD interfaces that adjust information presentation based on the driver’s cognitive traits and the current driving scenario.

## 2. Related Work and Research Hypotheses

### 2.1. Impact of AR-HUD Information Load on Cognitive Efficiency

The main benefit of AR-HUDs lies in merging virtual information with the real driving field of view [7], thereby reducing visual distraction and the cognitive load [8]. However, poorly designed displays can render the AR-HUD a new source of mental burden. Cognitive load theory (CLT) explains this: working memory is limited, and any task uses part of this capacity [9]. The external cognitive load—the load induced by information presentation methods and task organization [10]—is directly influenced by the AR-HUD information load.

Although AR-HUDs can improve awareness by overlaying virtual information onto the real world [11], simply adding more information is risky. A higher information load directly elevates the extraneous cognitive load. Malleable attentional resources theory further clarifies that, while drivers’ attentional resources can dynamically adjust, they possess clear upper limits [12]. When AR-HUD information nears or passes this limit, overload occurs, leading to slower reactions, errors, or missed prompts.

Current empirical research consistently indicates that information overload has become a primary risk in AR-HUD design. First, at the level of attention allocation, the information displayed on the AR-HUD interface competes visually with critical road elements, distracting the driver’s attention [13]. Moreover, when all information is highlighted simultaneously, it increases the likelihood of inattentional blindness, leading drivers to overlook critical elements even while maintaining their gaze on the road [14]. Secondly, this distraction rapidly translates into quantifiable increases in cognitive load and declines in behavioral performance. Research indicates that, under information-intensive or highly complex conditions, AR-HUD use significantly increases drivers’ cognitive loads, leading to longer reaction times and higher missed detection rates [15]. Too many visual prompts can also reduce accuracy in recognizing other road elements and lower situational awareness [7]. Finally, from the perspective of user experience and subjective perception, drivers clearly perceive the distraction effects caused by AR-HUDs [16]. This perceived distraction directly influences their evaluation and acceptance of the system [3]. This user-level evidence confirms that information overload is indeed present and poses a substantial threat to the system’s usability and safety.

Therefore, AR-HUD design should not aim for maximum information but for a dynamic, personalized load that prevents overload.

### 2.2. Impact of Driving Scenario on Cognitive Efficiency

Driving scenarios shape visual cognition, with complexity coming from the road layout, traffic density, and other environmental factors [17]. Complex driving scenarios increase the cognitive load, reduce driving efficiency, and threaten safety [18]. In demanding situations such as hidden exits or intersections, drivers exhibit significantly elevated skin conductance levels and pupil diameters, indicating an increased cognitive load [19]. EEG studies also show that the cognitive load rises with scene complexity—from expressways (low) to rural roads (medium) to urban settings (high)—and directly affects driving performance [20].

The fundamental cognitive demands of driving scenarios also impact the design effectiveness and safety of AR-HUDs. The unexpected overlay of virtual information onto the real environment may exacerbate inattentional blindness, causing drivers to overlook critical hazards on the actual road while focusing on AR prompts [21]. Environmental complexity can even outweigh potential AR-HUD benefits, lowering situational awareness [22]. Designs that match the scene’s cognitive needs improve reaction efficiency and safety; mismatched designs increase cognitive conflict [23]. For example, an AR-HUD that uses scene-aware reasoning can offload cognitive work, improve guidance accuracy, and lower overload risks compared to non-adaptive designs [24].

Thus, driving scenarios systematically constrain AR-HUD design. Optimized interfaces must treat the scenario as a key parameter to ensure efficient visual cognition and safety across different environments.

### 2.3. Impact of Working Memory Capacity on Cognitive Efficiency

WMC reflects a person’s ability to maintain and process information during tasks [25]. It supports visual perception, from early visual processing [26] to target search [27] and final decision-making [28]. Working memory acts as a mental workspace [29], storing visual features [30,31], building object representations [32], and linking to long-term knowledge [33]. Individual WMC differences directly moderate the overall efficiency of visual cognitive processes.

This influence is reflected in gaze behavior, largely driven by attentional control. High-WMC individuals excel not because they store more but because they control their attention better [34,35,36]. This allows for more effective focus and interference suppression, leading to faster visual search and better performance in cluttered settings [37].

These benefits extend to real-world tasks. In data-rich jobs, high-WMC individuals demonstrate greater resilience and efficiency in information processing [38,39]. Thus, WMC shapes how people process information in complex visual settings, mainly through attentional control. Therefore, studying AR-HUD design through the lens of WMC is essential in understanding driver behavior and creating safer, more user-friendly systems.

### 2.4. Research Questions

Existing research has illuminated the independent roles of the driving scenario, WMC, and AR-HUD information load in shaping driver performance. However, it remains unclear how these three factors interact to influence AR-HUD cognitive efficiency jointly. Specifically, the following research questions guide this study:

How do driving scenarios, WMC, and the AR-HUD information load affect AR-HUD cognitive performance and eye movements?

Is there a significant interaction among the driving scenario, WMC, and information load in determining cognitive efficiency?

How should the amount of information displayed on the AR-HUD be designed for different driving scenarios and working memory capacities?

To address these questions, this study systematically investigates the three-way interaction among the working memory capacity, driving scenario, and information load and applies these findings to develop design strategies for AR-HUD information presentation, thereby providing empirical support for adaptive interfaces. Eye tracking and behavioral data are used to quantify the cognitive load and to reveal differences in information-processing strategies between high- and low-WMC groups. Specifically, participants were first grouped by WMC using an automated operation span task. Then, a driving simulator equipped with eye tracking recorded reaction times, gaze patterns, and pupil sizes across three driving scenarios and six levels of visual information. By quantifying how the scenario demands, individual capacity, and interface load interact to influence cognitive efficiency, this research aims to establish practical guidelines for the design of adaptive AR-HUD systems.

## 3. Method for Quantifying Information Load of AR-HUD Interfaces

According to CLT [40,41], the intrinsic load is determined by task significance and decision urgency, while the extraneous load arises from the presentation style. In AR-HUD systems, these two types interact. Therefore, the information load of an AR-HUD interface depends on both the number of elements and their content attributes and presentation modalities. Quantifying the information load requires the simultaneous consideration of the information’s functional category and presentation form.

### 3.1. Evaluation Model Construction Based on AHP

The analytic hierarchy process (AHP) [42] was used to construct a multi-criteria evaluation model. The goal layer was “comprehensive information load weight of AR-HUD visual information”. Two criterion layers were defined: “information functional category” and “information presentation form”.

The information functional category reflects the priority of information in driving tasks. In-vehicle AR-HUD information was classified into two functional categories. Driving-related information comprised three subcategories: cue/warning (requiring a timely response), navigation overview (providing route context), and situational information (showing the immediate surroundings). Status information covered the vehicle status and road condition/event status. The information presentation form reflects the visual expression of information, including augmented reality (AR) graphics, icons, and text/numbers.

Fourteen AR-HUD experts were invited to perform pairwise comparisons of elements at each level. After excluding samples that failed the consistency test (CR < 0.1), 10 valid evaluation forms were retained, and the final weights for each level are shown in Table 1.

### 3.2. Comprehensive Information Load of a Single Information Unit

The comprehensive information load of a single AR-HUD visual information unit is obtained by linearly summing its functional category weight and presentation form weight.

(1) $$W_{i}=w_{f}\cdot w_{\left\{fc\right\}\left(i\right)}+w_{p}\cdot w_{\left\{pc\right\}\left(i\right)}$$

where $w_{f}$ and $w_{p}$ are the criterion layer weights for the information functional category and presentation form, respectively; $w_{\left\{fc\right\}\left(i\right)}$ is the local weight of the functional category to which information unit i belongs; and $w_{\left\{pc\right\}\left(i\right)}$ is the local weight of the presentation form to which information unit i belongs. $W_{i}$ is the comprehensive weight score based on the AHP weight system, whose actual range is determined by the set of functional category and presentation form weights defined in this study, and its value does not necessarily fall within the [0,1] interval. A larger $W_{i}$ indicates a higher relative contribution of that information unit to the driver’s cognitive resource consumption in the interface.

Based on the above formula, the comprehensive information load weights for each possible combination of information functional category and presentation form were calculated, as shown in Table 2.

### 3.3. Aggregation and Quantitative Control of Total Interface Load

The overall information load of an AR-HUD interface is defined as the sum of the loads of each independent visual unit within the interface. Suppose that an AR-HUD interface contains n independent visual information units, and the information load of the *i*-th unit is $W_{i}$; then, the total interface load $I_{\mathrm{total}}$ is defined as

(2) $$I_{\mathrm{total}}=\sum_{i=1}^{n}W_{i}$$

This formula reflects the cumulative effect of each information unit on the driver’s cognitive resource consumption. A larger $I_{\mathrm{total}}$ indicates higher overall cognitive resource occupation and a higher information load. Based on the calculation results for individual information units, this formula enables the estimation of the total information load for different interface configurations and allows for quantitative control of the total interface load $I_{\mathrm{total}}$ by adding or removing information units or adjusting their functional categories and presentation forms.

In summary, this section quantifies the weights of functional categories and presentation forms of AR-HUD visual information through the AHP and expert judgment. It establishes a comprehensive information load model based on weighted accumulation, providing a methodological foundation for the quantitative assessment of the interface information load.

## 4. Experimental Research

### 4.1. Experiment 1: Working Memory Capacity (WMC) Test

#### 4.1.1. Participants

Forty participants were recruited to complete the WMC test. Participants ranged in age from 20 to 27 years (mean = 23.35, SD = 1.594), with 18 males and 22 females in the sample.

#### 4.1.2. Materials and Methods

**Figure 1:**
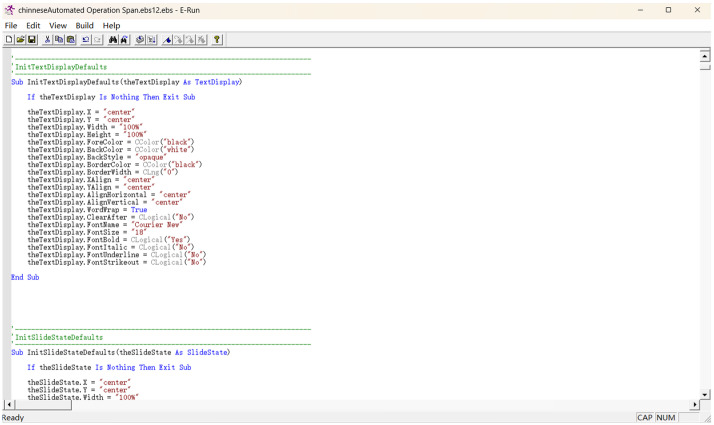
Actual software screenshot of automated operation span (AOSPAN) task.

WMC was assessed using an automated operation span (AOSPAN) task [43] implemented in E-Prime 1.1.4.1; the software interface is shown in Figure 1. The experiment comprised a practice phase and a formal test phase. During the practice phase, participants sequentially completed three tasks to understand the rules: (1) a letter recall task; (2) a math operation task; and (3) a dual-task combining letter recall and math operation. The formal testing phase comprised 15 sets of dual-task trials, each containing 5 math operations and their corresponding 5 letters, for a total of 75 math operations and 75 letters. Participants were required to judge the correctness of each operation while simultaneously memorizing the presented letters, recalling them sequentially at the end of each trial. Consistent with previous studies [44,45], the total span score served as the indicator of the individual WMC, with scores ranging from 0 to 75. Higher scores indicate greater WMC.

#### 4.1.3. Experimental Results

Scores from 40 participants in the WMC test were analyzed using SPSS 27. The scores ranged from 34 to 75 (mean = 60.15, median = 63.50, SD = 10.647). A Shapiro–Wilk test indicated that the data were not normally distributed (*p* < 0.001).

### 4.2. Experiment 2: AR-HUD Visual Cognition Experiment

#### 4.2.1. Participants

G*Power 3.1 was used to estimate the required sample size for the experiment. With the significance level (*α*) set at 0.05, an effect size (*f*) of 0.25, and statistical power of 0.95, the calculation indicated a minimum total sample size of 14 participants. To account for potential attrition and enhance the reliability of the findings, 32 participants were ultimately recruited from the initial cohort of Experiment 1. The top 16 high-scoring individuals (mean = 68.81, SD = 3.082) were assigned to the high-WMC group, while the 16 lowest scorers (mean = 50.00, SD = 9.494) formed the low-WMC group. The final sample consisted of 32 participants (17 female, 15 male), with a mean age of 23.19 years (SD = 1.533), as shown in Table 3. All participants held valid motor vehicle driver’s licenses, had corrected visual acuity of 4.4 or higher, and had no color vision deficiencies. Before the experiment, all participants reviewed the study protocol and provided written informed consent. Participants received monetary compensation ranging from CNY 20 to 30 based on task accuracy.

#### 4.2.2. Apparatus

**Figure 2:**
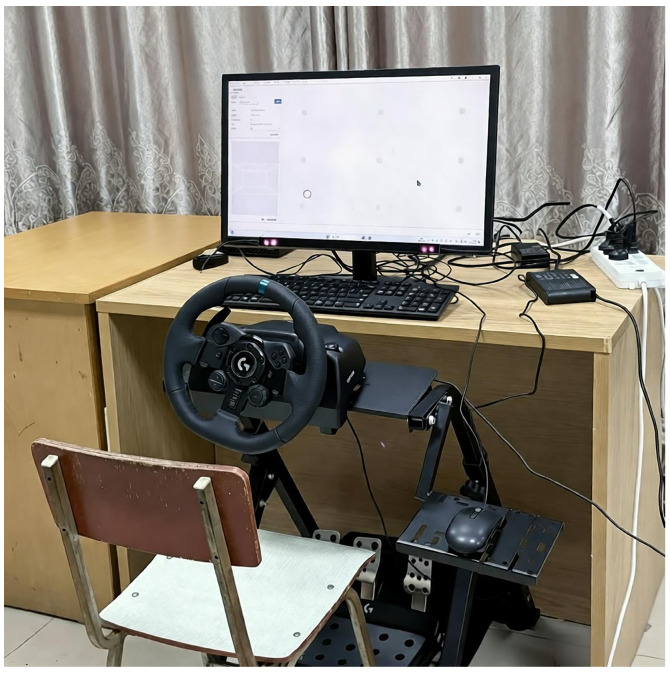
Driving simulator system equipment.

The experimental setup utilized a low-immersivity, desktop-based virtual reality (VR) driving simulation system (Figure 2). The driving simulator system consisted of a force-feedback steering wheel and a pedal set connected to a high-performance computer that generated the simulated driving environment. Within this VR environment, all visual stimuli—including the driving scenarios, the vehicle cockpit, and the AR-HUD graphical elements—were rendered in a unified 3D virtual space (Unity version 2022.3.53) and presented on a 24-inch display (1920 × 1080 pixels, 144 Hz refresh rate) from a fixed, forward-facing driver’s perspective. A Logitech G923 controller (Logitech, Lausanne, Switzerland) controlled the steering wheel and pedals. Data from the driving simulator were collected via the Logitech G HUB 2025.9 software. The driving scenario was set during the daytime with good visibility and dry road conditions. Eye movement data were recorded using a Tobii TX300 eye tracker (Tobii Technology AB, Danderyd, Sweden) at a sampling rate of 300 Hz.

#### 4.2.3. Experimental Materials

(1) Driving Scenario Design

Three distinct driving scenarios were designed for this experiment.

(A) Urban Intersection. Vehicle A (the ego vehicle) was traveling normally at 50 km/h on an urban road, following Vehicle B ahead, which was moving at 40 km/h. As they approached a signalized intersection, the need to make a left turn arose.
(B) Expressway. Vehicle A (the ego vehicle) was traveling at 90 km/h in the slow lane of an expressway. As it approached a section with multiple exits, the need to change lanes to the right to enter an off-ramp was triggered.
(C) Construction Zone. Vehicle A (the ego vehicle) was traveling at 50 km/h through an urban construction zone. A temporary traffic light on the left showed green, while a pedestrian appeared on the right, crossing from right to left. To maintain safe driving, the need to decelerate and stop arose.

**Figure 3:**
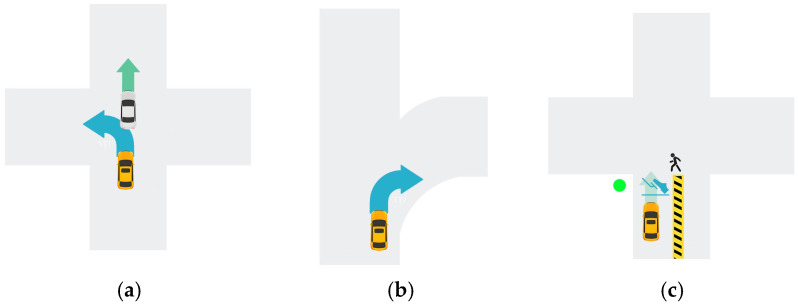
Driving scenario design. (**a**) Urban intersection. Subject task: Perform a safe left turn. (**b**) Expressway. Subject task: Perform a lane change to the right. (**c**) Construction zone. Subject task: Slow down to stop and give way. The yellow vehicle is the ego vehicle; Blue icons/arrows indicate the required driving maneuvers.

The specific driving scenarios are illustrated in Figure 3. All scenarios were built as interactive 3D environments in the low-immersivity VR system and presented as video stimuli from a fixed first-person driving perspective. This experiment adopted the method from prior studies of using trigger points to activate experimental tasks [46,47]. Specifically, when the driver’s vehicle reached a pre-set distance, the system automatically triggered AR-HUD navigation prompts and corresponding driving tasks.

(2) Design of Visual Information Levels for AR-HUD Interfaces

Based on the AHP weight matrix established in Table 2, this study aimed to design six visual information levels (VILs). Given that the determination of AHP weights relies on expert judgment, which inherently involves uncertainty even after consistency testing [48], a sensitivity analysis was first conducted to determine the feasible fluctuation range of the total information load for each VIL, ensuring the cross-scenario consistency of the total load across different driving scenarios in the subsequent design.

**Figure 4:**
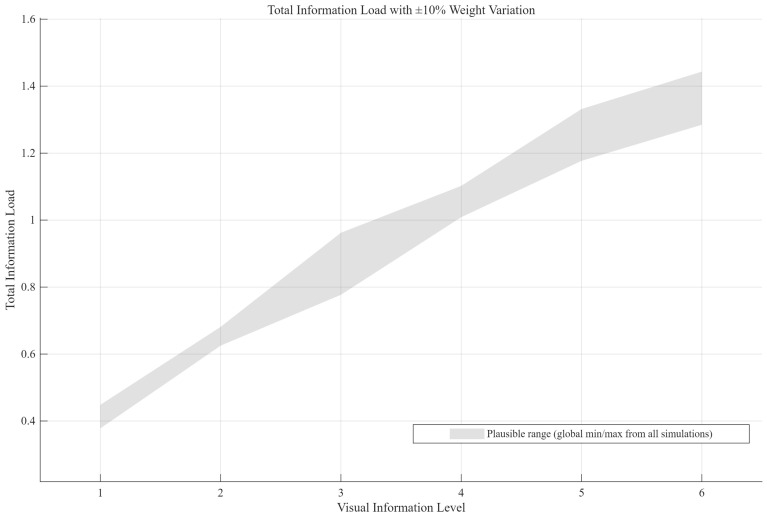
Total information load fluctuation range for each visual information level (VIL) under sensitivity analysis.

Following the standard approach to AHP sensitivity analysis [49], the local weights within each criterion were allowed to vary randomly within ±10% of their original values, and 1000 Monte Carlo iterations were performed while maintaining the normalization constraint that weights summed to one within each criterion. To ensure reproducibility, the random number generator was initialized with a fixed seed (rng (42)). For each VIL, the total information load depended on the combination of information elements at that level. During the sensitivity analysis, the total load was calculated for all plausible information combinations (i.e., each functional category in its common presentation forms). The 3000 simulated values obtained for each VIL (3 scenarios × 1000 iterations) were then aggregated. The minimum and maximum values were used to define the feasible fluctuation range of the total information load for that VIL (extreme value method), as shown in Table 4 and Figure 4. This range covered all possible extreme cases under uncertainty.

**Figure 5:**
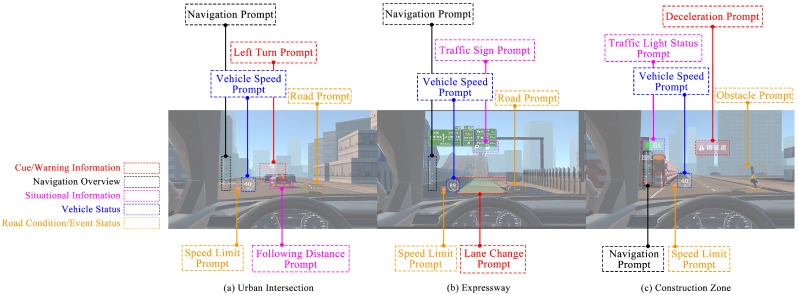
AR-HUD interface screenshots for the six VILs across the three driving scenarios. The image presents horizontally concatenated first-person driver views from the virtual reality (VR) environment, with overlaid callout boxes indicating the functional categories of the displayed information elements. The AR navigation graphic was spatially registered to the road surface, simulating the conformal display characteristic of real AR-HUD systems. The experimental stimuli were presented in Chinese to the participants. Some AR-HUD information elements (e.g., route guidance, distance-to-destination, front road name, “Slow Down” warning) therefore appear in Chinese in the relevant screenshots.

**Figure 6:**
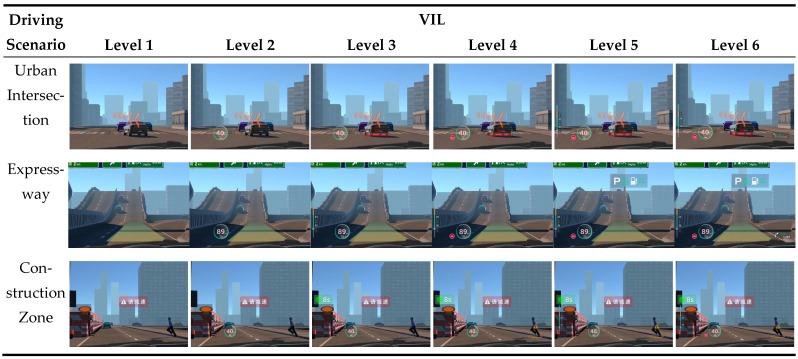
First-person VR view screenshots for all 18 experimental conditions. Rows represent the three driving scenarios (urban intersection, expressway, construction zone); columns represent the six VILs (1 to 6). Chinese text appears in the stimuli for the same reason as noted in Figure 5.

Based on the above quantitative results, this study designed six VILs, each composed of information elements with different functional categories and presentation forms, as shown in Table 5 and Figure 5. To provide a complete reference for the experimental stimuli, Figure 6 presents the first-person VR screenshots for all 18 experimental conditions (3 driving scenarios × 6 VILs). Each row corresponds to one driving scenario, and each column corresponds to one VIL, allowing a direct visual comparison of how the AR-HUD interface complexity increased across information levels within and across scenarios. Operationally, when the driver’s vehicle reached the pre-set trigger points within the virtual environment, the system automatically displayed the designated combination of AR graphics, icons, and text/number elements on the windshield area.

**Figure 7:**
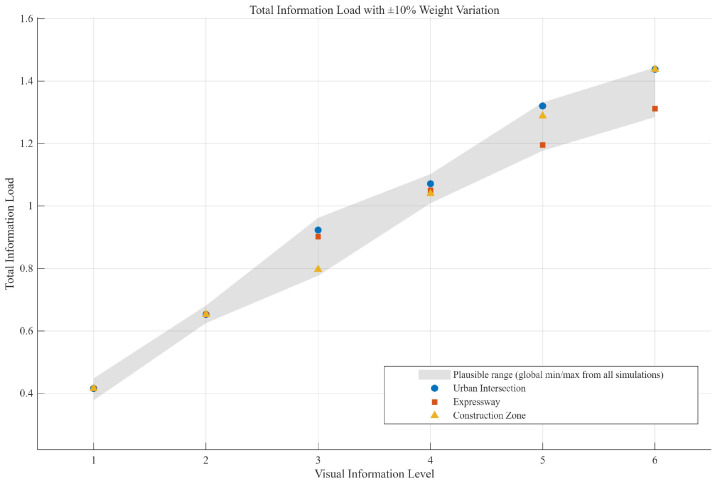
Actual total information load for each VIL across driving scenarios with sensitivity analysis range.

According to the aggregation model, the total information load for each scenario and each level was calculated, with the results presented in Table 6 and Figure 7. The actual calculated loads for all three scenarios fell within the fluctuation range, indicating that the six VILs exhibited cross-scenario consistency in information load and that the experimental design was robust against reasonable variations in expert judgment.

Key optical parameters were set as follows: the field of view (FOV) was 13° (horizontal) × 5° (vertical) [50], and the virtual image distance (VID) was 7.5 m [51]. Based on this configuration, the theoretical dimensions of the system’s virtual image were 170.9 cm (width) × 65.6 cm (height). Ultimately, the projection module presented an effective display area of 52 cm (width) × 24 cm (height) on the windshield, occupying approximately 40% of the total frontal projection area.

#### 4.2.4. Experimental Design and Procedure

This study included three independent variables: WMC (high vs. low), VIL (six levels: 1–6), and the driving scenario (urban intersection, expressway, and construction zone). To minimize the influence of individual differences on the experimental results, a 6 × 3 within-subjects design was employed. The 18 experimental conditions (3 driving scenarios × 6 VILs) were implemented as 15 s video stimuli and administered using the Ergo LAB 3.16.13 stimulus presentation software. To control for order effects while ensuring exposure to all conditions, the experiment was structured into two blocks. Each block contained all 18 conditions, yielding 36 trials per participant. Within each block, the trial sequence was pseudo-randomized under the constraint that no identical condition appeared on two consecutive trials, thereby preventing the immediate repetition of the same combination of scenario and information load. The randomization procedure was independently executed for each participant using Ergo LAB’s built-in randomized list generator, resulting in a unique viewing sequence for each participant. A 1 min rest period was provided between blocks to mitigate fatigue. Each participant completed all 36 trials, with the entire session taking approximately 15 min.

Before the formal experiment, all participants completed familiarization trials for the three scenarios to ensure that they understood the cue information that they needed to attend to during simulated driving. This phase involved watching simulated driving videos with the VIL set to Level 1, allowing participants to also practice operating the simulator controls.

**Figure 8:**
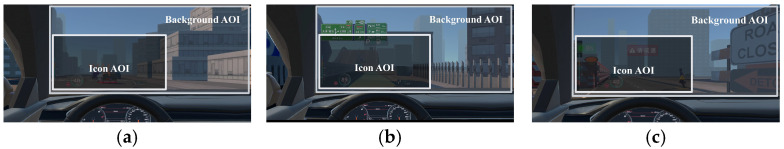
Definition of areas of interest (AOI) across driving scenarios: (**a**) urban intersection; (**b**) expressway; (**c**) construction zone. Chinese text appears in the stimuli for the same reason as noted in Figure 5.

Throughout the experiment, participants’ eye movement data were systematically recorded using an eye tracking system to evaluate visual perception characteristics and quantitatively assess the identification efficiency. As illustrated in Figure 8, two distinct areas of interest (AOIs) were defined for the AR-HUD interface: the icon AOI and the background AOI.

**Figure 9:**
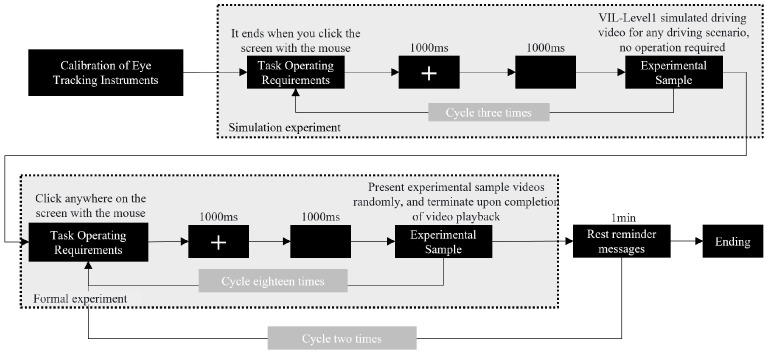
Experimental flowchart.

Before the experiment, the seat was adjusted to ensure that the participant’s line of sight was parallel to the center of the screen, with a viewing distance of 50–60 cm. After calibrating the eye tracker, on-screen instructions were presented. Participants then sequentially completed 3 practice trials followed by 36 formal experimental trials. As shown in Figure 9, the formal trials were divided into two blocks, each containing 18 trials presented in a randomized order. Each trial began with a fixation cross “+” displayed for 1000 ms, followed by a black screen for 1000 ms, after which an experimental stimulus was randomly presented. When participants observed a driving operation cue appearing in the scenario, they were required to respond promptly using the driving simulator controls. A 1 min rest period was provided after each block. The experiment was concluded after all formal trials were completed.

#### 4.2.5. Data Collection and Processing

Data were collected from all 32 participants. Two categories of metrics were obtained: behavioral and oculometric. The behavioral metric was the reaction time (RT), defined as the interval between the appearance of an AR-HUD cue and the driver’s correct operational response (steering or braking). Oculometric indicators within the icon AOI were extracted: the total fixation duration (TFD), average fixation duration (AFD), and average pupil diameter (APD).

At the trial level, trials were excluded from all analyses if they met any of the following criteria: (1) RT < 100 ms, indicating an anticipatory or premature response; (2) RT > 3000 ms, indicating non-response or attentional disengagement; or (3) the participant executed an incorrect driving operation (e.g., braking when steering was required). At the participant level, the entire dataset for 5 participants was removed because their gaze tracking ratio fell below 80%. After applying all exclusion criteria, behavioral and eye tracking analyses were conducted on 27 participants, resulting in a data retention rate of 84.38%. For each of the 18 experimental conditions, we retained between 51 and 54 valid trials out of a possible maximum of 54 (27 participants × 2 repetitions), and this range was consistent across all three driving scenarios.

These metrics reflect distinct cognitive processes: RT directly reflects the speed at which a driver processes information and makes decisions [52]; TFD and AFD collectively indicate the allocation of visual attention resources and the depth of information processing (longer durations suggest greater cognitive resources allocated to and higher engagement with the current area) [53]; APD has been widely recognized as a reliable physiological indicator reflecting the cognitive load and neural resource consumption [54]. All data were processed in SPSS and analyzed using a generalized linear model (GLM).

## 5. Results

### 5.1. Data Analysis

A GLM was fitted with WMC, VIL, and the driving scenario as fixed effects and the participant ID as a random effect. The resulting main effects table is presented in Table 7.

### 5.2. Reaction Time (RT)

As shown in Table 7, the main effects of both WMC (*χ*^2^ = 5.434, *p* = 0.020 < 0.05) and the driving scenario (*χ*^2^ = 491.861, *p* < 0.001) reached statistical significance. For WMC, the low-WMC group demonstrated significantly longer RTs than the high-WMC group (*β* = 0.029, *p* = 0.020 < 0.05). Regarding the driving scenarios, RTs were significantly faster in both the urban intersection (*β* = −0.247, *p* < 0.001) and the expressway (*β* = −0.314, *p* < 0.001) than in the construction zone.

Additionally, a significant interaction effect was observed between VIL and the driving scenario (*χ*^2^ = 19.097, *p* = 0.039 < 0.05). As shown in Table 8, across all VILs, RTs in the construction zone were significantly longer than those in both the expressway and urban intersection (*p* < 0.001). No significant differences were observed between the urban intersection and the expressway at most VILs (*p* > 0.05), except at Level 4, where a significant difference emerged (*p* < 0.001), with significantly longer RTs in the urban intersection than in the expressway.

**Figure 10:**
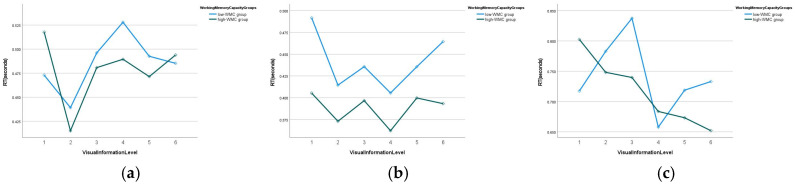
Estimated marginal means of reaction time by WMC group and VIL across driving scenarios: (**a**) urban intersection; (**b**) expressway; (**c**) construction zone.

The three-way interaction effect among VIL, WMC, and the driving scenario on the RT did not reach statistical significance (*χ*^2^ = 8.029, *p* = 0.626). The estimated marginal means of the RT across VILs, WMC groups, and driving scenarios are presented in Figure 10. Consistent with the non-significant three-way interaction, the pattern showed that the low-WMC group generally reacted more slowly than the high-WMC group. However, individual cell-level differences were non-significant for most VIL × scenario combinations, and occasional reversals reflected sampling variability.

### 5.3. Total Fixation Duration (TFD) for the Icon AOI

As shown in Table 7, the main effects of WMC (*χ*^2^ = 29.188, *p* < 0.001), VIL (*χ*^2^ = 1233.830, *p* < 0.001), and the driving scenario (*χ*^2^ = 159.624, *p* < 0.001) were highly significant. Regarding WMC, the low-WMC group showed a significantly shorter TFD than the high-WMC group (*β* = −0.749, *p* < 0.001). For VIL, TFD showed an increasing trend with higher VILs: those at Levels 1, 2, and 3 were all significantly lower than for the baseline Level 6 (*p* < 0.001), while Levels 4 and 5 showed no significant difference from Level 6 (*p* > 0.05). In terms of driving scenarios, the urban intersection required the longest TFD, which was significantly longer than in the construction zone (*β* = 1.230, *p* < 0.001). The expressway showed the shortest TFD, which differed significantly from that in the construction zone (*β* = −0.906, *p* < 0.001).

Regarding interaction effects, significant interactions were observed between WMC and VIL (*χ*^2^ = 17.996, *p* = 0.003 < 0.05), between VIL and the driving scenario (*χ*^2^ = 7.609, *p* = 0.022 < 0.05), and between WMC and the driving scenario (*χ*^2^ = 844.948, *p* < 0.001). Specifically, at lower VILs (Levels 2 and 3), the high-WMC group demonstrated significantly longer TFDs than the low-WMC group (*p* < 0.001). However, when the VIL reached Level 4 or higher, the difference in TFD between WMC groups was no longer significant (*p* > 0.05). Furthermore, in both the urban intersection and the construction zone, TFD showed a clear upward trend with rising VILs, being particularly significant between Levels 1 and 3 (*p* < 0.001). In contrast, on the expressway, TFD remained consistently low across all VILs, with minimal fluctuations, and showed no significant differences across most levels (*p* > 0.05).

**Figure 11:**
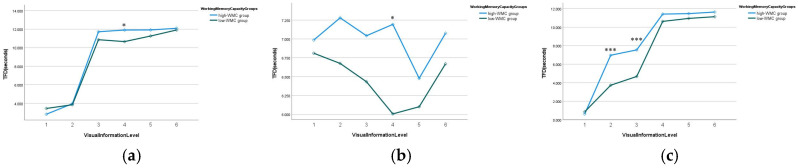
Estimated marginal means of TFD on icon AOI by WMC group and VIL across driving scenarios: (**a**) urban intersection; (**b**) expressway; (**c**) construction zone. * *p* < 0.05. *** *p* < 0.001 for pairwise comparisons between WMC groups.

Additionally, a significant three-way interaction among VIL, WMC, and the driving scenario was observed (*χ*^2^ = 19.341, *p* = 0.036 < 0.05). To decompose this interaction, simple effects of WMC were examined at each VIL × scenario combination, with effect sizes quantified by Cohen’s *d* (Table 9). Specifically, the interaction pattern between WMC and VIL varied across different driving scenarios. As shown in Figure 11, at the urban intersection, the TFD for both the high- and low-WMC groups increased steadily as the VIL increased. However, when the VIL reached Level 4 or higher, the curves for both groups ran parallel, and the influence of VIL on TFD was weakened. Simple-effects analyses revealed that the between-group difference in TFD was not significant at VILs 1–3 (*p*s > 0.05, *d* < 0.25), reached marginal significance at VIL 4 (*p* = 0.033, *d* = 0.34), and became non-significant again at VILs 5–6 (*p*s > 0.05, *d* < 0.19). On the expressway, the TFD of the high-WMC group reached its lowest point at Level 5 and then gradually increased, whereas the low-WMC group reached its lowest point at Level 4 before rising. Across most VILs, the difference in TFD between the high- and low-WMC groups did not reach statistical significance (all *p*s > 0.05), except for VIL 4, where a small but significant effect emerged (*p* = 0.043, *d* = 0.33). At the construction zone, the interaction pattern resembled that at the urban intersection but was more complex. At low VILs (Levels 2 and 3), the high-WMC group exhibited significantly longer TFDs than the low-WMC group (*p* < 0.001), with large effect sizes (VIL 2: *d* = 0.89; VIL 3: *d* = 0.79). As the VIL increased to Level 4 and above, the difference in TFD between the two groups gradually diminished and became non-significant (all *p*s > 0.05, *d* range: 0.14–0.22).

### 5.4. Average Fixation Duration (AFD) for the Icon AOI

As shown in Table 7, the main effects of WMC (*χ*^2^ = 5.476, *p* = 0.019 < 0.05), VIL (*χ*^2^ = 128.631, *p* < 0.001), and the driving scenario (*χ*^2^ = 63.451, *p* < 0.001) were all highly significant. Regarding WMC, the high-WMC group demonstrated a significantly longer AFD than the low-WMC group (*β* = 0.064, *p* = 0.019 < 0.05). Regarding VILs, the AFD at Level 1 was significantly shorter than at Level 6 (*β* = −0.339, *p* < 0.001), whereas, at Level 3, it was significantly longer than at Level 6 (*β* = 0.185, *p* < 0.001). In terms of driving scenarios, the AFD in the urban intersection was significantly longer than in both the expressway and construction zone (*p* < 0.001).

Regarding interaction effects, a significant interaction between VIL and the driving scenario was observed (*χ*^2^ = 156.822, *p* < 0.001). As shown in Table 10, at the lowest VIL (Level 1), no significant difference was observed between the urban intersection and expressway (*p* = 0.900 > 0.05). However, at both medium (Levels 2 and 3) and high (Levels 4 and 5) VILs, the urban intersection exhibited significantly longer AFDs than the expressway (*p* < 0.001). Interestingly, at the highest VIL (Level 6), the difference between these two scenarios became marginal (*p* = 0.052 > 0.05). More pronounced variations were observed in the construction zone compared to the other scenarios. At low (Level 1) and high (Levels 4 to 6) VILs, the construction zone showed significantly shorter AFDs than both the urban intersection and expressway. Conversely, at medium VILs (Levels 2 and 3), the AFD in the construction zone was significantly longer than in both the urban intersection and expressway (*p* < 0.05).

The three-way interaction effect among VIL, WMC, and the driving scenario on AFD did not reach statistical significance (*χ*^2^ = 14.470, *p* = 0.153).

### 5.5. Average Pupil Diameter (APD)

The GLM effect test revealed that neither the VIL (*χ*^2^ = 0.393, *p* > 0.05) nor the driving scenario (*χ*^2^ = 0.105, *p* > 0.05) showed statistical significance. However, a significant main effect was observed for WMC (*χ*^2^ = 32.966, *p* < 0.001). As shown in Table 7, the low-WMC group demonstrated a significantly larger APD than the high-WMC group (*β* = 0.3690, *p* < 0.001).

Regarding interaction effects, no significant interactions were observed between WMC and VIL, between WMC and the driving scenario, or between VIL and the driving scenario (all *p*s > 0.05). Similarly, there was no interaction effect among the three factors (*χ*^2^ = 0.183, *p* > 0.05).

## 6. Discussion

Through simulated driving experiments, this study systematically examined the effects of WMC, VIL, and the driving scenario on drivers’ operational performance and visual attention behavior. Unlike previous studies that mostly focused on the main effects of single factors, this study integrated CLT [9] and attentional control theory (ACT) [34] to reveal a significant three-way interaction among WMC, VIL, and the driving scenario (TFD: *χ*^2^ = 19.341, *p* = 0.036). This finding extends the linear “information load–performance” relationship in CLT to a nonlinear framework characterized by the interaction of “individual–scenario–load,” indicating that the moderating effect of WMC on the AR-HUD information processing efficiency is not constant but systematically constrained by the cognitive demands of the driving scenario. The following discussion addresses three main aspects: individual cognitive mechanisms, scenario moderation mechanisms, and the theoretical implications of the three-way interaction.

### 6.1. Cognitive Mechanisms Underlying the Moderating Role of Working Memory Capacity (WMC) in Information Processing Efficiency

The experimental results indicate that high-WMC drivers exhibited faster RTs across all experimental conditions, consistent with the prediction of ACT [34] that high-WMC individuals have an advantage in attentional control (RT: *β* = −0.029, *p* = 0.020 < 0.05) [55]. However, an in-depth analysis of the gaze behavior revealed a more complex cognitive mechanism: the performance advantage of high-WMC individuals did not stem from less gaze investment; rather, they exhibited significantly longer TFDs and AFDs (TFD: *β* = 0.749, *p* < 0.001; AFD: *β* = 0.064, *p* = 0.019 < 0.05). This seemingly “inefficient” gaze pattern actually reflects effective attentional allocation [37]. High-WMC drivers proactively prolonged their fixations on AR-HUD information to integrate multiple sources and build more accurate situational representations, thus translating greater visual investment into faster decisions. This finding extends the interpretation of ACT [34] from “resource differences” to “strategy differences”: the advantage of high-WMC individuals lies not only in possessing more cognitive resources but also in their strategic ability to decide “when to invest and how much to invest,” achieving optimal resource allocation.

Pupil diameter data provide direct physiological evidence for this interpretation. The high-WMC group maintained a significantly smaller APD across all conditions (*β* = −0.3690, *p* < 0.001), indicating that they consumed fewer neural resources to complete the same information processing tasks [56], reflecting their inherently higher efficiency in information processing [57]. When the VIL reached Level 4 and above, the low-WMC group showed no significant difference in TFD compared with the high-WMC group (*p* > 0.05), but their RTs did not accelerate correspondingly. This suggests that the visual cognitive function of low-WMC individuals changes with the load level: under low loads (VIL 1–3), visual cognition primarily engages in information capture to quickly locate key information; under high loads (VIL ≥ 4), their visual cognition shifts to passive maintenance—although fixating, they struggle to achieve spatial binding and semantic integration across information units. Research by Kosachenko et al. (2023) [58] indicates that pupil dilation serves as an early warning sign of working memory overload, consistent with the significantly larger APD observed in the low-WMC group in this study. This finding provides new empirical support for CLT [9]: the extraneous cognitive load (determined by information presentation) and intrinsic cognitive load (determined by task complexity) can be mitigated in high-WMC individuals through strategic resource allocation, whereas, in low-WMC individuals, the coupling of these two types of load rapidly triggers cognitive overload.

**Figure 12:**
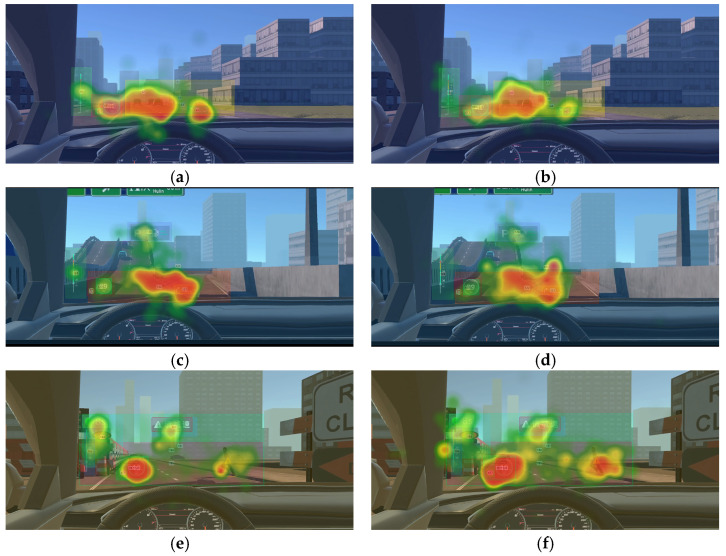
Comparison of gaze heatmaps on the icon AOI between WMC groups across driving scenarios: (**a**) urban intersection/high-WMC group; (**b**) urban intersection/low-WMC group; (**c**) expressway/high-WMC group; (**d**) expressway/low-WMC group; (**e**) construction zone/high-WMC group; (**f**) construction zone/low-WMC group. Warmer colors (yellow to red) indicate higher visual attention, while cooler colors (green) indicate lower visual attention. Chinese text appears in the stimuli for the same reason as noted in Figure 5.

As shown in Figure 12, the gaze heatmaps provide intuitive visual evidence supporting the above interpretation. Under the same driving scenario, the gaze trajectory in the high-WMC group was broader and more continuous, covering multiple key elements within the AR-HUD information area; in contrast, the gaze of the low-WMC group was more concentrated and fragmented, tending to dwell on a single information point for a longer time before jumping to other areas, lacking a continuous information-scanning pattern. This difference indicates that high-WMC individuals adopt a systematic scanning strategy, integrating dispersed information into a holistic cognitive schema. In contrast, low-WMC individuals adopt a point-focused strategy, struggling to establish effective connections among multiple information sources.

### 6.2. Moderating Effect of Driving Scenario on Information Load Effects

The results reveal a significant moderating effect of the driving scenario on VIL effects (RT: *χ*^2^ = 19.097, *p* = 0.039 < 0.05; AFD: *χ*^2^ = 156.822, *p* < 0.001), but this moderating effect is not simply a matter of “greater information load effects in complex scenarios”; rather, it exhibits a complex, nonlinear pattern. To understand this phenomenon, this paper interprets the concept of “driving scenario cognitive demand” through the lens of “element interactivity” from CLT [9]. Element interactivity refers to the degree to which multiple information elements must be processed simultaneously to achieve understanding—higher interactivity implies a higher intrinsic cognitive load. Accordingly, the three scenarios in this study—expressway, urban intersection, and construction zone—correspond to low, medium, and high element interactivity, respectively.

First, the expressway represents a low-element-interactivity scenario. In such scenarios, the driving task is highly structured (lane keeping, constant speed), and elements such as speed and lane position can be processed relatively independently. Drivers can form stable attentional templates based on experience, thereby reducing the reliance on AR-HUD information. As seen in the data analysis, on the expressway, the TFD remained consistently low across all VILs, with no significant differences (*p* > 0.05), and drivers showed the lowest sensitivity to VIL changes. The review by Winkler and Soleimani (2025) [4] also notes that the cognitive load effects of AR-HUD are design-sensitive and scenario-dependent. In other words, when the element interactivity of the scenario itself is low, an increase in the AR-HUD information load does not significantly interfere with drivers’ attentional allocation.

Second, the urban intersection represents a medium-element-interactivity scenario. In such scenarios, drivers need to integrate multi-source information from both AR-HUD navigation and the real road environment (traffic signals, traffic flow status) to make complex decisions [5]. Unlike in the expressway, gaze investment (TFD, AFD) at the urban intersection increased significantly with rising VILs, and this gaze investment translated into faster reaction times (e.g., the RT in the urban intersection was significantly shorter than in the construction zone). This suggests that, under medium levels of element interactivity, drivers can compensate for the cognitive demands imposed by the information load by actively increasing their gaze investment, thereby adopting an adaptive strategy of “exchanging fixation for speed.”

Finally, the construction zone represents a high-element-interactivity scenario. In such scenarios, the driving task is sudden and irregular, and environmental elements such as temporary traffic lights, crossing pedestrians, and lane change instructions are highly coupled [47]. In well-structured roads, the consistency of geometric design elements provides a predictable spatial reference frame [59], and standardized lane markings and intersection designs further reinforce this predictability. AR-HUD spatial markers—such as navigation graphics and situational information icons—rely on this framework to function as anticipatory cues that align with drivers’ experience-based expectations. The construction zone, however, disrupts exactly this foundation: temporary traffic controls, irregular lane boundaries, and unexpected pedestrian paths remove the stable spatial reference that gives these markers their predictive value. Stripped of this reference, spatial markers no longer support prediction; instead, they compete for the very attentional resources that drivers need to track dynamically changing hazards [60]. This explains why the construction zone exhibited the slowest RTs across all VILs, and even moderate TFD investment failed to translate into decision-making advantages (the RT in the construction zone was significantly longer than in other scenarios; the TFD increased with the VIL in the construction zone, but the RT did not accelerate).

**Figure 13:**
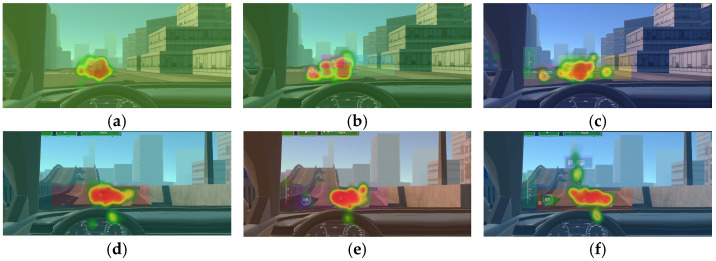

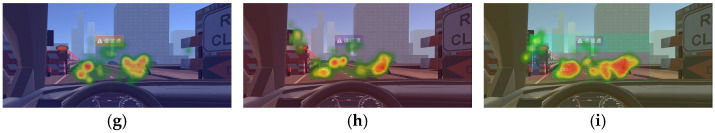
Gaze heatmaps on the icon AOI across three driving scenarios at VILs 1, 3, and 6: (**a**) urban intersection/Level 1; (**b**) urban intersection/Level 3; (**c**) urban intersection/Level 6; (**d**) expressway/Level 1; (**e**) expressway/Level 3; (**f**) expressway/Level 6; (**g**) construction zone/Level 1; (**h**) construction zone/Level 3; (**i**) construction zone/Level 6. Warmer colors (yellow to red) indicate higher visual attention, while cooler colors (green) indicate lower visual attention. Chinese text appears in the stimuli for the same reason as noted in Figure 5.

Gaze heatmaps across different driving scenarios and VILs (Figure 13) provide intuitive evidence. In driving scenarios with a medium-to-high cognitive demand, the gaze distribution across the AR-HUD information area became more dispersed as the VIL increased. In contrast, in low-demand scenarios, the gaze distribution remained relatively concentrated. This finding has important implications for AR-HUD design. In high-element-interactivity scenarios, increasing the information load not only fails to improve the decision quality but may even increase safety risks by diverting drivers’ attention from real-world sudden events [14].

### 6.3. Three-Way Interaction: Boundary Conditions of WMC Moderation

The significant three-way interaction effect on TFD (*χ*^2^ = 19.341, *p* = 0.036 < 0.05) constitutes the core finding of this study. It reveals clear boundary conditions for WMC’s moderating effect: once the VIL exceeded Level 4, the between-group TFD difference became small or non-significant across all three scenarios (*d* < 0.22 at VIL ≥ 5).

This pattern can be explained by the interplay between the two mechanisms identified in the preceding sections: the element interactivity of the driving scenario (from CLT [9]) and the attentional control advantage of high-WMC drivers (from ACT [34]). Element interactivity determines the scenario’s intrinsic cognitive load. Attentional control determines whether drivers can strategically allocate resources to handle additional information. Their joint operation explains why WMC differences appeared, disappeared, and reappeared across conditions.

In the low-interactivity expressway, the intrinsic load was minimal. Even with increasing AR-HUD information, the total cognitive demand remained below the capacity ceiling for both WMC groups. Consequently, WMC differences were submerged—the task was too simple for the high-WMC group’s attentional control advantage to matter (all *p*s > 0.05, *d*: 0.05–0.33). In the medium-interactivity urban intersection, the total demand entered a range in which strategic attentional control became operational, granting high-WMC drivers greater tolerance for information load (VIL 4–6). In the high-interactivity construction zone, the intrinsic load was already extreme. Adding AR-HUD information pushed the total demand toward even the high-WMC drivers’ capacity limits, eroding their advantage at higher VILs (VIL ≥ 4: all *p*s > 0.05, *d*: 0.14–0.22). This explains why the construction zone showed the steepest increase in cognitive load and why increasing VILs failed to translate into faster responses.

This interplay also explains the differential critical points for overload. Low-WMC drivers with limited attentional control consistently reached overload at VIL 4. High-WMC drivers delayed overload to VILs 5–6 in low- and medium-demand scenarios through strategic allocation. However, in the high-interactivity construction zone, even their superior control was insufficient, shifting their critical point earlier.

In previous studies, Luke et al. (2018) [61] found that high-WMC individuals exhibited significantly longer average fixation durations during scene viewing, while Redden et al. (2023) [37] reported shorter reaction times and lower omission rates under dual-task conditions. The present study extends these findings by demonstrating that high-WMC drivers achieve both longer fixations and faster reactions within the same task, confirming deeper information integration during each fixation. Critically, this advantage is contingent on the driving scenario’s element interactivity—a boundary condition for WMC’s predictive power [34].

### 6.4. AR-HUD Design Strategies for Individual Differences and Scenario Adaptation

Based on the above findings, this study proposes the following design strategies for the AR-HUD information load. It should be noted that these strategies are derived from a young sample aged 20–27 years, and their applicability to older drivers requires further validation.

(1) Strategies for high-WMC drivers: In low-element-interactivity scenarios (expressway) and medium-element-interactivity scenarios (urban intersection), richer information content (VILs 4–5) can be presented to support the decision-making advantages brought by their deep processing strategies. In high-element-interactivity scenarios (construction zone), the information load should be controlled at a moderate level (VILs 3–4) to avoid disrupting their strategic processing patterns due to information overload. It should be noted that, even at VIL 5, the gaze behavior of the high-WMC group already showed signs of increased resource consumption; therefore, it is recommended to set the upper limit of the information load at VIL 5 to reserve a cognitive margin for unexpected events.
(2) Strategies for low-WMC drivers: The principle of “less is more” should be followed in all scenarios. In high-element-interactivity scenarios, only the minimum necessary information (VILs 1–2) should be presented. In medium-demand scenarios, a moderately low information load (VILs 2–3) is appropriate. Only in low-demand scenarios can the information load be slightly increased (VIL 4). The core goal is to prevent low-WMC drivers from entering a passive state of attention, ensuring that their limited cognitive resources are used effectively used for real-world perception and decision-making.
(3) Because excessive compression of the information load may lead to decreased situational awareness, practical applications need to seek a balance between avoiding overload and maintaining situational awareness. Future systems adopting dynamic adaptive strategies should fine-tune the information load based on drivers’ real-time eye tracking metrics (e.g., TFD, APD) rather than relying solely on static thresholds.

### 6.5. Research Limitations and Future Directions

While this study reveals the three-way interaction among WMC, the driving scenario, and the information load, it has several limitations that future research should address.

First, the experimental paradigm and simulator environment may have reduced the cognitive demands compared with real-world driving. The experiment employed a low-immersivity, desktop-based VR approach—specifically, a passive video-viewing paradigm in a driving simulator—which introduces two related but distinct limitations. The passive viewing paradigm involved no active vehicle control (steering, lane keeping, collision avoidance), meaning that there was no resource competition between the driving task itself and AR-HUD information processing. Consequently, our estimated safe VIL thresholds may be overestimated for real driving. More generally, the simulator environment lacked genuine risk perception, emotional arousal, a physical presence, and real consequences for errors. This may have reduced the overall cognitive and affective load, potentially leading to the underestimation of the moderating role of working memory capacity in high-risk scenarios. Practitioners should therefore adopt more conservative loads (e.g., reduce by 1–2 VIL levels), and future research should validate the present findings under active driving conditions (high-fidelity simulators or real roads) with realistic hazard consequences and multisensory feedback (e.g., vibration, motion).

Second, the participant age range was limited to 20–27 years, which constrains the generalizability of the findings. This limitation is particularly consequential because older drivers—the population most likely to struggle with high information loads—were excluded. Existing research has documented an age-related decline in working memory capacity and processing speed. Given that this study has already shown that, even among young adults, individuals with lower WMC experience cognitive overload at markedly lower VIL thresholds, it is reasonable to anticipate that the VIL thresholds identified here for both high- and low-WMC groups may be overestimated relative to those suitable for older drivers. Therefore, the current thresholds should not be directly generalized to an older population without further validation. Future research should systematically recruit participants across the adult lifespan to examine how age and WMC jointly moderate tolerance for AR-HUD information loads. Such work is essential in establishing age-inclusive design standards that protect the most vulnerable road users.

Third, this study primarily relied on pupil diameters and eye movement behavior to assess the cognitive load. Within the eye tracking domain, future studies could incorporate the blink rate to better capture visual fatigue and attentional disengagement. Beyond ocular measures, multimodal physiological indicators such as heart rate variability (HRV) should be integrated with electroencephalography (EEG) data to reflect emotional stress and autonomic regulation. Combining these richer eye tracking and physiological signals would allow the more precise identification of the neurophysiological critical points of cognitive overload.

Fourth, although this study quantified the information load using AHP weights and verified the robustness of the results through sensitivity analysis, factors such as the semantic relevance and spatial layout of information elements across different driving scenarios were not controlled for. Future research should manipulate the semantic relevance and spatial distribution of information elements while keeping the total information load constant to explore the moderating effects of WMC on information structural characteristics.

## 7. Conclusions

This study investigated how driving scenarios, WMC, and AR-HUD information loads jointly affect cognitive performance in AR-HUD interfaces. First, high- and low-WMC participants were screened with an automated operation span task (AOSPAN). A driving simulator equipped with eye tracking was then used to record the RT, gaze behavior, and pupil diameter across three scenarios (urban intersection, expressway, construction zone) and six levels of visual information.

The results revealed significant three-way interactions among the driving scenario, WMC, and VIL. High-WMC drivers translated longer fixation times into performance advantages and tolerated VILs of 4–6, whereas low-WMC drivers showed cognitive overload beyond VIL 4 and required VILs of 2–3. In medium- or high-demand scenarios, increasing VILs exacerbated the load; in the low-demand expressway, drivers were less sensitive to load changes. The system should therefore set personalized VIL thresholds based on WMC and adjust the presentation dynamically to match the scenario demands, ensuring safe and efficient situational awareness.

These findings underscore the need to integrate WMC and scenario complexity when specifying the amount of AR-HUD information. Individual cognitive capacity and environmental demand must be treated jointly in cognitive–ergonomics research. This study provides both theoretical grounding and empirical evidence for personalized AR-HUD design in intelligent vehicles. It should be noted, however, that the specific VIL thresholds identified here were derived from a young adult sample (aged 20–27 years) and should not be directly generalized to older drivers without further validation. For safety-critical applications, more conservative thresholds are advisable until age-diverse data become available. Future work should explore other determinants of visual efficiency and develop real-time VIL adaptation strategies tailored to WMC and driving scenarios. Such advances would enhance situational awareness and decision quality in complex environments, thereby guiding the development of next-generation AR-HUD systems.

## Figures and Tables

**Table 1:** Weight summary of criterion and subcriterion layers for augmented reality head-up display (AR-HUD) visual information load.

Criterion Layer	Weight	Subcriterion Layer	Local Weight	Global Weight
Information Functional Category	0.598	Cue/Warning	0.349	0.209
		Navigation Overview	0.280	0.167
		Situational Information	0.105	0.063
		Vehicle Status	0.207	0.124
		Road Condition/Event	0.059	0.035
Information Presentation Form	0.402	augmented reality (AR) Graphic	0.515	0.207
		Icon	0.282	0.113
		Text/Number	0.203	0.082

**Table 2:** Weight matrix of comprehensive information load for single AR-HUD visual information units.

	Information Presentation Form	AR Graphic	Icon	Text/Number
Information Functional Category				
Cue/Warning		0.416	0.322	0.291
Navigation Overview		0.374	0.280	0.249
Situational Information		0.270	0.176	0.145
Vehicle Status		0.331	0.237	0.206
Road Condition/Event		0.242	0.148	0.117

**Table 3:** Subject information.

Working Memory Capacity (WMC)	Age Mean	Score Minimum	Score Maximum	Score Average	Score Standard (SD)
High	22.81	65	75	68.81	3.082
Low	23.56	34	61	50.00	9.494

**Table 4:** Fluctuation range for each level (extreme value method).

Level	Minimum	Maximum
1	0.37795	0.44825
2	0.62508	0.68116
3	0.777	0.96219
4	1.0088	1.1022
5	1.177	1.3317
6	1.2848	1.4434

**Table 5:** VIL solution table for different driving scenarios.

Driving Scenario	VIL	Driving-Related Information			Status Information	
		Cue/Warning Information	Navigation Overview	Situational Information	Vehicle Status	Road Condition/ Event Status
Urban Intersection	Level 1	1A	0	0	0	0
	Level 2	1A	0	0	1I	0
	Level 3	1A	0	1A	1I	0
	Level 4	1A	0	1A	1I	1I
	Level 5	1A	1T	1A	1I	1I
	Level 6	1A	1T	1A	1I	1I1T
Expressway	Level 1	1A	0	0	0	0
	Level 2	1A	0	0	1I	0
	Level 3	1A	1T	0	1I	0
	Level 4	1A	1T	0	1I	1I
	Level 5	1A	1T	1T	1I	1I
	Level 6	1A	1T	1T	1I	1I1T
Construction Zone	Level 1	1A	0	0	0	0
	Level 2	1A	0	0	1I	0
	Level 3	1A	0	1T	1I	0
	Level 4	1A	0	1T	1I	1A
	Level 5	1A	1T	1T	1I	1A
	Level 6	1A	1T	1T	1I	1A1I

Non-zero values indicate the presence of the corresponding information element; a value of 0 indicates that it is not provided. Multiple suffixes (e.g., 1I1T) indicate that this category contains multiple pieces of information, each presented in a distinct display form.

**Table 6:** Total information load of AR-HUD interface under various scenarios and levels.

Level	Urban Intersection	Expressway	Construction Zone
1	0.416	0.416	0.416
2	0.653	0.653	0.653
3	0.923	0.902	0.798
4	1.071	1.05	1.04
5	1.32	1.195	1.289
6	1.437	1.312	1.437

**Table 7:** Results of the generalized linear model for main effects.

Analyzed Variable	Independent Variable	*χ* ^2^	Degrees of Freedom	*p*
Reaction Time (s)	Intercept	7923.054	1	0.000
	WMC	5.434	1	0.020
	VIL	8.540	5	0.129
	Driving Scenario	491.861	2	0.000
Total Fixation Duration (s)	Intercept	12,488.589	1	0.000
	WMC	29.188	1	0.000
	VIL	1233.830	5	0.000
	Driving Scenario	159.624	2	0.000
Average Fixation Duration (s)	Intercept	2397.465	1	0.000
	WMC	5.476	1	0.019
	VIL	128.631	5	0.000
	Driving Scenario	63.451	2	0.000
Average Pupil Diameter (mm)	Intercept	16,338.313	1	0.000
	WMC	32.966	1	0.000
	VIL	0.393	5	0.996
	Driving Scenario	0.105	2	0.949

**Table 8:** Pairwise comparisons of reaction times across driving scenarios at different VILs.

VIL	Comparison Between Complexity Groups (Group 1–Group 2)	*β*	Standard Error	*p*	95%CI	
					Lower Limit	Upper Limit
Level 1	Urban Intersection–Expressway	0.052	0.041	0.209	−0.029	0.132
	Urban Intersection–Construction Zone	−0.266 ^a^	0.041	0.000	−0.347	−0.185
	Expressway–Construction Zone	−0.317 ^a^	0.041	0.000	−0.399	−0.236
Level 2	Urban Intersection–Expressway	0.033	0.0333	0.311	−0.032	0.099
	Urban Intersection–Construction Zone	−0.338 ^a^	0.0331	0.000	−0.403	−0.273
	Expressway–Construction Zone	−0.372 ^a^	0.0331	0.000	−0.437	−0.307
Level 3	Urban Intersection–Expressway	0.073	0.042	0.083	−0.010	0.156
	Urban Intersection–Construction Zone	−0.297 ^a^	0.042	0.000	−0.380	−0.215
	Expressway–Construction Zone	−0.371 ^a^	0.042	0.000	−0.453	−0.288
Level 4	Urban Intersection–Expressway	0.125 ^a^	0.036	0.000	0.055	0.194
	Urban Intersection–Construction Zone	−0.164 ^a^	0.036	0.000	−0.234	−0.094
	Expressway–Construction Zone	−0.289 ^a^	0.036	0.000	−0.359	−0.218
Level 5	Urban Intersection–Expressway	0.065	0.035	0.066	−0.004	0.133
	Urban Intersection–Construction Zone	−0.214 ^a^	0.035	0.000	−0.282	−0.145
	Expressway–Construction Zone	−0.278 ^a^	0.035	0.000	−0.346	−0.210
Level 6	Urban Intersection–Expressway	0.062	0.032	0.052	−0.001	0.124
	Urban Intersection–Construction Zone	−0.202 ^a^	0.032	0.000	−0.264	−0.140
	Expressway–Construction Zone	−0.263 ^a^	0.032	0.0000	−0.325	−0.202

^a^ The significance level is 0.05.

**Table 9:** Simple effects of WMC on total fixation duration (TFD) at each VIL × driving scenario combination.

Scenario	VIL	High-WMC TFD (SE)	Low-WMC TFD (SE)	Mean Diff.	*p*	Cohen’s *d*
Urban Intersection	1	2.82 (0.41)	3.45 (0.42)	−0.62	0.287	−0.17
	2	3.96 (0.41)	3.85 (0.42)	0.11	0.848	0.03
	3	11.72 (0.41)	10.86 (0.42)	0.86	0.143	0.24
	4	11.90 (0.41)	10.66 (0.42)	1.25	0.033	0.34
	5	11.91 (0.41)	11.26 (0.42)	0.66	0.263	0.18
	6	12.09 (0.41)	11.91 (0.42)	0.18	0.759	0.05
Expressway	1	6.99 (0.41)	6.81 (0.42)	0.17	0.765	0.05
	2	7.28 (0.41)	6.68 (0.42)	0.6	0.302	0.17
	3	7.04 (0.41)	6.43 (0.42)	0.61	0.297	0.17
	4	7.19 (0.41)	6.01 (0.42)	1.19	0.043	0.33
	5	6.48 (0.41)	6.10 (0.42)	0.38	0.520	0.1
	6	7.08 (0.41)	6.67 (0.46)	0.41	0.508	0.11
Construction Zone	1	0.62 (0.42)	0.85 (0.42)	−0.24	0.693	−0.06
	2	6.97 (0.41)	3.73 (0.42)	3.24	<0.001	0.89
	3	7.55 (0.41)	4.67 (0.42)	2.87	<0.001	0.79
	4	11.42 (0.41)	10.63 (0.42)	0.79	0.179	0.22
	5	11.47 (0.41)	10.95 (0.42)	0.52	0.375	0.14
	6	11.64 (0.42)	11.13 (0.41)	0.5	0.391	0.14

**Table 10:** Pairwise comparisons of average fixation duration across driving scenarios at different VILs.

VIL	Comparison Between Complexity Groups (Group 1–Group 2)	*β*	Standard Error	*p*	95%CI	
					Lower Limit	Upper Limit
Level 1	Urban Intersection–Expressway	0.007	0.059	0.900	−0.108	0.123
	Urban Intersection–Construction Zone	0.227 ^a^	0.060	0.000	0.110	0.344
	Expressway–Construction Zone	0.219 ^a^	0.060	0.000	0.102	0.336
Level 2	Urban Intersection–Expressway	0.269 ^a^	0.098	0.006	0.076	0.462
	Urban Intersection–Construction Zone	−0.236 ^a^	0.098	0.016	−0.429	−0.043
	Expressway–Construction Zone	−0.505 ^a^	0.098	0.000	−0.698	−0.312
Level 3	Urban Intersection–Expressway	0.284 ^a^	0.104	0.006	0.081	0.488
	Urban Intersection–Construction Zone	−0.238 ^a^	0.104	0.022	−0.441	−0.035
	Expressway–Construction Zone	−0.523 ^a^	0.104	0.000	−0.726	−0.319
Level 4	Urban Intersection–Expressway	0.253 ^a^	0.090	0.005	0.077	0.428
	Urban Intersection–Construction Zone	0.350 ^a^	0.090	0.000	0.174	0.525
	Expressway–Construction Zone	0.097	0.090	0.279	−0.079	0.273
Level 5	Urban Intersection–Expressway	0.301 ^a^	0.061	0.000	0.181	0.421
	Urban Intersection–Construction Zone	0.623 ^a^	0.061	0.000	0.503	0.744
	Expressway–Construction Zone	0.323 ^a^	0.061	0.000	0.202	0.443
Level 6	Urban Intersection–Expressway	0.305	0.077	0.052	0.153	0.456
	Urban Intersection–Construction Zone	0.617 ^a^	0.077	0.000	0.465	0.768
	Expressway–Construction Zone	0.312 ^a^	0.077	0.000	0.160	0.463

^a^ The significance level is 0.05.

## Data Availability

The original contributions presented in the study are included in the article; further inquiries can be directed to the corresponding author.

## Abbreviations

The following abbreviations are used in this manuscript:

AR-HUD	Augmented Reality Head-Up Display
WMC	Working Memory Capacity
VR	Virtual Reality
CLT	Cognitive Load Theory
ACT	Attentional Control Theory
AHP	Analytic Hierarchy Process
AOSPAN	Automated Operation Span
VIL	Visual Information Level
AR	Augmented Reality
FOV	Field of View
VID	Virtual Image Distance
AOI	Area of Interest
RT	Reaction Time
TFD	Total Fixation Duration
AFD	Average Fixation Duration
APD	Average Pupil Diameter
GLM	Generalized Linear Model
HRV	Heart Rate Variability
EEG	Electroencephalography

## References

[1] Gabbard J.L., Fitch G.M., Kim H. (2014). Behind the glass: Driver challenges and opportunities for AR automotive applications. Proc. IEEE.

[2] Feierle A., Schlichtherle F., Bengler K. (2021). Augmented reality head-up display: A visual support during malfunctions in partially automated driving?. IEEE Trans. Intell. Transp. Syst..

[3] Li X., Rong J., Li Z., Zhao X., Zhang Y. (2022). Modeling drivers’ acceptance of augmented reality head-up display in connected environment. Displays.

[4] Winkler M., Soleimani M. (2025). A review of augmented reality heads up display in vehicles: Effectiveness, application, and safety. Int. J. Hum.–Comput. Interact..

[5] Zhou C., Luo Y., Kaner J. (2025). Exploring the impact of display types of information about autonomous driving in semi-autonomous vehicles on drivers’ situation awareness and take-over performance under different driving scenarios. PLoS ONE.

[6] Draheim C., Pak R., Draheim A.A., Engle R.W. (2022). The role of attention control in complex real-world tasks. Psychon. Bull. Rev..

[7] Kim H., Gabbard J.L. (2022). Assessing distraction potential of augmented reality head-up displays for vehicle drivers. Hum. Factors.

[8] Korentsides J., St Clair A., Dyapa M., Chaparro A. (2022). Head-Up Displays: Analysis of automotive use considerations. Hum. Factors Access. Assist. Technol..

[9] Sweller J. (1988). Cognitive load during problem solving: Effects on learning. Cogn. Sci..

[10] Sweller J., Chandler P. (1994). Why some material is difficult to learn. Cogn. Instr..

[11] Bauerfeind K., Drüke J., Schneider J., Haar A., Bendewald L., Baumann M. (2021). Navigating with augmented reality–how does it affect drivers’ mental load?. Appl. Ergon..

[12] Young M.S., Stanton N.A. (2002). Malleable attentional resources theory: A new explanation for the effects of mental underload on performance. Hum. Factors.

[13] Li J., Chen K., Chen M. (2025). The Influence of Information Redundancy on Driving Behavior and Psychological Responses Under Different Fog and Risk Conditions: An Analysis of AR-HUD Interface Designs. Appl. Sci..

[14] Wang L., Li H., Guo M., Chen Y. (2022). The effects of dynamic complexity on drivers’ secondary task scanning behavior under a car-following scenario. Int. J. Environ. Res. Public Health.

[15] Maag C., Schömig N., Naujoks F., Karl I., Keinath A., Neukum A. (2023). Measuring workload effects of augmented reality head-up displays using detection response task. Transp. Res. Part F Traffic Psychol. Behav..

[16] Eyraud R., Zibetti E., Baccino T. (2015). Allocation of visual attention while driving with simulated augmented reality. Transp. Res. Part F Traffic Psychol. Behav..

[17] Tian H., Deng T., Yan H. (2022). Driving as well as on a sunny day? predicting driver’s fixation in rainy weather conditions via a dual-branch visual model. IEEE/CAA J. Autom. Sin..

[18] Wascher E., Alyan E., Karthaus M., Getzmann S., Arnau S., Reiser J.E. (2023). Tracking drivers’ minds: Continuous evaluation of mental load and cognitive processing in a realistic driving simulator scenario by means of the EEG. Heliyon.

[19] Nilsson E.J., Bärgman J., Ljung Aust M., Matthews G., Svanberg B. (2022). Let complexity bring clarity: A multidimensional assessment of cognitive load using physiological measures. Front. Neuroergon..

[20] Wascher E., Arnau S., Reiser J.E., Rudinger G., Karthaus M., Rinkenauer G., Dreger F., Getzmann S. (2019). Evaluating mental load during realistic driving simulations by means of round the ear electrodes. Front. Neurosci..

[21] Chen W., Song J., Wang Y., Wu C., Ma S., Wang D., Yang Z., Li H. (2023). Inattentional blindness to unexpected hazard in augmented reality head-up display assisted driving: The impact of the relative position between stimulus and augmented graph. Traffic Inj. Prev..

[22] Currano R., Park S.Y., Moore D.J., Lyons K., Sirkin D. Little road driving hud: Heads-up display complexity influences drivers’ perceptions of automated vehicles. Proceedings of the 2021 CHI Conference on Human Factors in Computing Systems.

[23] Zhu Q., Yu T., Jung E. (2025). Seeing and Hearing the Turn: Multimodal AR-HUD Navigation in Multi-Branch Road Scenarios. IEEE Access.

[24] Liang Y., Zheng P., Xia L. (2023). A visual reasoning-based approach for driving experience improvement in the AR-assisted head-up displays. Adv. Eng. Inform..

[25] Cowan N. (2017). The many faces of working memory and short-term storage. Psychon. Bull. Rev..

[26] Hellmann S., Zehetleitner M., Rausch M. (2023). Simultaneous modeling of choice, confidence, and response time in visual perception. Psychol. Rev..

[27] Soon C.S., Namburi P., Chee M.W. (2013). Preparatory patterns of neural activity predict visual category search speed. NeuroImage.

[28] Ratcliff R. (1978). A theory of memory retrieval. Psychol. Rev..

[29] Logie R.H., Della Sala S. (2003). Working memory as a mental workspace: Why activated long-term memory is not enough. Behav. Brain Sci..

[30] Sahakian A., Gayet S., Paffen C.L., Van der Stigchel S. (2025). The rise and fall of memories: Temporal dynamics of visual working memory. Mem. Cogn..

[31] Xu L., Sahakian A., Gayet S., Paffen C.L., Van der Stigchel S. (2025). Latent memory traces for prospective items in visual working memory. J. Exp. Psychol. Hum. Percept. Perform..

[32] Karlsen P.J., Allen R.J., Baddeley A.D., Hitch G.J. (2010). Binding across space and time in visual working memory. Mem. Cogn..

[33] Hirschstein Z., Aly M. (2023). Long-term memory and working memory compete and cooperate to guide attention. Atten. Percept. Psychophys..

[34] Engle R.W. (2002). Working memory capacity as executive attention. Curr. Dir. Psychol. Sci..

[35] Shipstead Z., Lindsey D.R., Marshall R.L., Engle R.W. (2014). The mechanisms of working memory capacity: Primary memory, secondary memory, and attention control. J. Mem. Lang..

[36] Zhao C., Vogel E.K. (2025). Working memory and attentional control abilities predict individual differences in visual long-term memory tasks. J. Mem. Lang..

[37] Redden R.S., Eady K., Klein R.M., Saint-Aubin J. (2023). Individual differences in working memory capacity and visual search while reading. Mem. Cogn..

[38] Bühner M., König C.J., Pick M., Krumm S. (2006). Working memory dimensions as differential predictors of the speed and error aspect of multitasking performance. Hum. Perform..

[39] Greenberg K., Zheng R., Gardner M., Orr M. (2021). Individual differences in visuospatial working memory capacity influence the modality effect. J. Comput. Assist. Learn..

[40] Sweller J. (2024). Cognitive load theory and individual differences. Learn. Individ. Differ..

[41] Krieglstein F., Beege M., Rey G.D., Sanchez-Stockhammer C., Schneider S. (2023). Development and validation of a theory-based questionnaire to measure different types of cognitive load. Educ. Psychol. Rev..

[42] Saaty T.L. (2008). Decision making with the analytic hierarchy process. Int. J. Serv. Sci..

[43] Unsworth N., Heitz R.P., Schrock J.C., Engle R.W. (2005). An automated version of the operation span task. Behav. Res. Methods.

[44] Conway A.R., Kane M.J., Bunting M.F., Hambrick D.Z., Wilhelm O., Engle R.W. (2005). Working memory span tasks: A methodological review and user’s guide. Psychon. Bull. Rev..

[45] Redick T.S., Broadway J.M., Meier M.E., Kuriakose P.S., Unsworth N., Kane M.J., Engle R.W. (2012). Measuring working memory capacity with automated complex span tasks. Eur. J. Psychol. Assess..

[46] Winkler S., Kazazi J., Vollrath M. (2018). How to warn drivers in various safety-critical situations–Different strategies, different reactions. Accid. Anal. Prev..

[47] Duan K., Yan X., Li X., Hang J. (2023). Improving drivers’ merging performance in work zone using an in-vehicle audio warning. Transp. Res. Part F Traffic Psychol. Behav..

[48] Masuda T., Fujii K. (1987). Sensitivity Analyses of Priorities Used in the Analytic Hierarchy Process (AHP). Trans. Soc. Instrum. Control Eng..

[49] Dhurkari R.K. (2023). Improving the prescriptive power of analytic hierarchy process. IEEE Trans. Eng. Manag..

[50] Fan R., Wei S., Ji H., Qian Z., Tan H., Mo Y., Ma D. (2023). Automated design of freeform imaging systems for automotive heads-up display applications. Opt. Express.

[51] Zhou C., Qiao W., Hua J., Chen L. (2024). Automotive augmented reality head-up displays. Micromachines.

[52] Niu Y.F., Yue G., Gao Y., Xue C.Q., Zhang Y.T., Yang L.X. (2020). Improving eye–computer interaction interface design: Ergonomic investigations of the optimum target size and gaze-triggering dwell time. J. Eye Mov. Res..

[53] Ren G., Zhao X., Lin Z., Xu W. (2019). Research on the visual cognition patterns of exit guide sign viewing on freeway interchanges. Adv. Mech. Eng..

[54] Bitkina O.V., Park J., Kim H.K. (2021). The ability of eye-tracking metrics to classify and predict the perceived driving workload. Int. J. Ind. Ergon..

[55] Li K., Yuan W., Yannis G., Wu F., Wang C. (2025). Investigating the impact of in-vehicle warning information complexity on drivers: The role of working memory capacity and cognitive load. Accid. Anal. Prev..

[56] Orti R., Iachini T., D’Agostino E., Ruotolo F., Ruggiero G. (2025). Cognitive load in switching between egocentric and allocentric spatial frames of reference: A pupillometry study. Sci. Rep..

[57] Zhang Y., Shao J., Qin L., Zhan Y., Zhao X., Geng M., Chen B. (2024). Semantic distance of icons: Impact on user cognitive performance and a new model for semantic distance classification. Int. J. Ind. Ergon..

[58] Kosachenko A.I., Kasanov D., Kotyusov A.I., Pavlov Y.G. (2023). EEG and pupillometric signatures of working memory overload. Psychophysiology.

[59] Afghari A.P., Vos J., Farah H., Papadimitriou E. (2023). “I did not see that coming”: A latent variable structural equation model for understanding the effect of road predictability on crashes along horizontal curves. Accid. Anal. Prev..

[60] Horrey W.J., Wickens C.D., Consalus K.P. (2006). Modeling drivers’ visual attention allocation while interacting with in-vehicle technologies. J. Exp. Psychol. Appl..

[61] Luke S.G., Darowski E.S., Gale S.D. (2018). Predicting eye-movement characteristics across multiple tasks from working memory and executive control. Mem. Cogn..

